# The effect of hamstring tightness on intraoperative extension gap in posterior stabilized total knee arthroplasty

**DOI:** 10.1038/s41598-021-83221-0

**Published:** 2021-02-11

**Authors:** Byung Woo Cho, Hyuck Min Kwon, Koo Yeon Lee, Kwan Kyu Park, Ick Hwan Yang, Woo-Suk Lee

**Affiliations:** 1 Department of Orthopaedic Surgery, Gangnam Severance Hospital, Yonsei University College of Medicine, 211 Eonju-ro, Gangnam-gu, Seoul, Republic of Korea; 2 Department of Orthopaedic Surgery, Severance Hospital, Yonsei University College of Medicine, 50-1 Yonsei-ro, Seodaemun-gu, Seoul, Republic of Korea

**Keywords:** Anatomy, Diseases, Medical research

## Abstract

This study aimed to determine the factors related to intraoperative extension gap (EG) in patients who underwent posterior-stabilized total knee arthroplasty (TKA). A total of 106 TKAs in 84 patients were retrospectively reviewed. Only patients who underwent the same method of bone resection were included consecutively. Bilateral popliteal angle (BPA) was used as an indicator of hamstring tightness. EG and extension space angle were measured using an offset type tensor. The associations between patient variables and EG were analyzed using multivariable linear regression and Pearson's correlation coefficients. The average EG was 12.9 ± 2.1 mm, and the average extension space angle was 2.8° ± 3.2°. BPA was greater than flexion contracture in most cases (94.3%), and no difference was found in only six cases (5.7%). According to multivariable linear regression analysis which was conducted after modifying the BPA into a categorical variable by 5°, EG was correlated with BPA (p < 0.001). Pearson’s correlation coefficient between EG and BPA was − 0.674 (p < 0.001). No other factors were significantly correlated with intraoperative EG. The present study found that popliteal angle is a different entity from flexion contracture, and that it is a predictable factor for EG in osteoarthritis patients. Smaller BPAs led to larger EG in patients who underwent the same degree of bone resection.

## Introduction

The size of proper extension gap (EG) and balance with the flexion gap are essential for achieving successful clinical outcomes in total knee arthroplasty (TKA)^1^. However, unexpected large EG can occur, even if the surgery is performed in the same way. The EG comprises the distance between distal femur and proximal tibia, as well as the laxity of soft tissue, including collateral ligaments and posterior structures of the knee. Since bone resection level can be controlled by the surgeon, unexpected gap differences between patients mainly occur due to the soft tissue laxity. When the knee joint is extended in the posterior stabilized TKA setting, cruciate ligaments are resected and the extensor mechanism is loose, leading the posterior structures of the knee and collateral ligaments to mainly affect the soft tissue laxity and the EG.

The anatomy of the posterior knee consists of a complicated network of dynamic and static stabilizers. Among them, the posterior semimembranosus complex was described as one of the major anatomical structures in the posterior aspect of the knee, according to LaPrade et al.^2^. Moreover, many studies have reported releasing hamstring musculature as a technique for adjusting the EG^3,4^. Taken together, it can be considered that the hamstring musculature is one of the major structures that could affect soft tissue laxity around the extended knee. If the hamstring tightness, which reflects the laxity of posterior structures of the knee, can be predicted in advance, the EG can be expected, and additional procedures or unnecessary bone resection can be avoided. However, to the best of our knowledge, the method for predicting the hamstring tightness and EG in elderly patients undergoing TKA is not yet known^5^. Base on this idea, we hypothesized that the EG can be predicted by measuring the hamstring tightness in elderly patients undergoing TKA. Therefore, the aim of this study was to determine whether factors such as hamstring tightness were related to intraoperative extension gap in patients who underwent the same amount of bone resection.

## Materials and methods

### Patient recruitment

**Figure 1:**
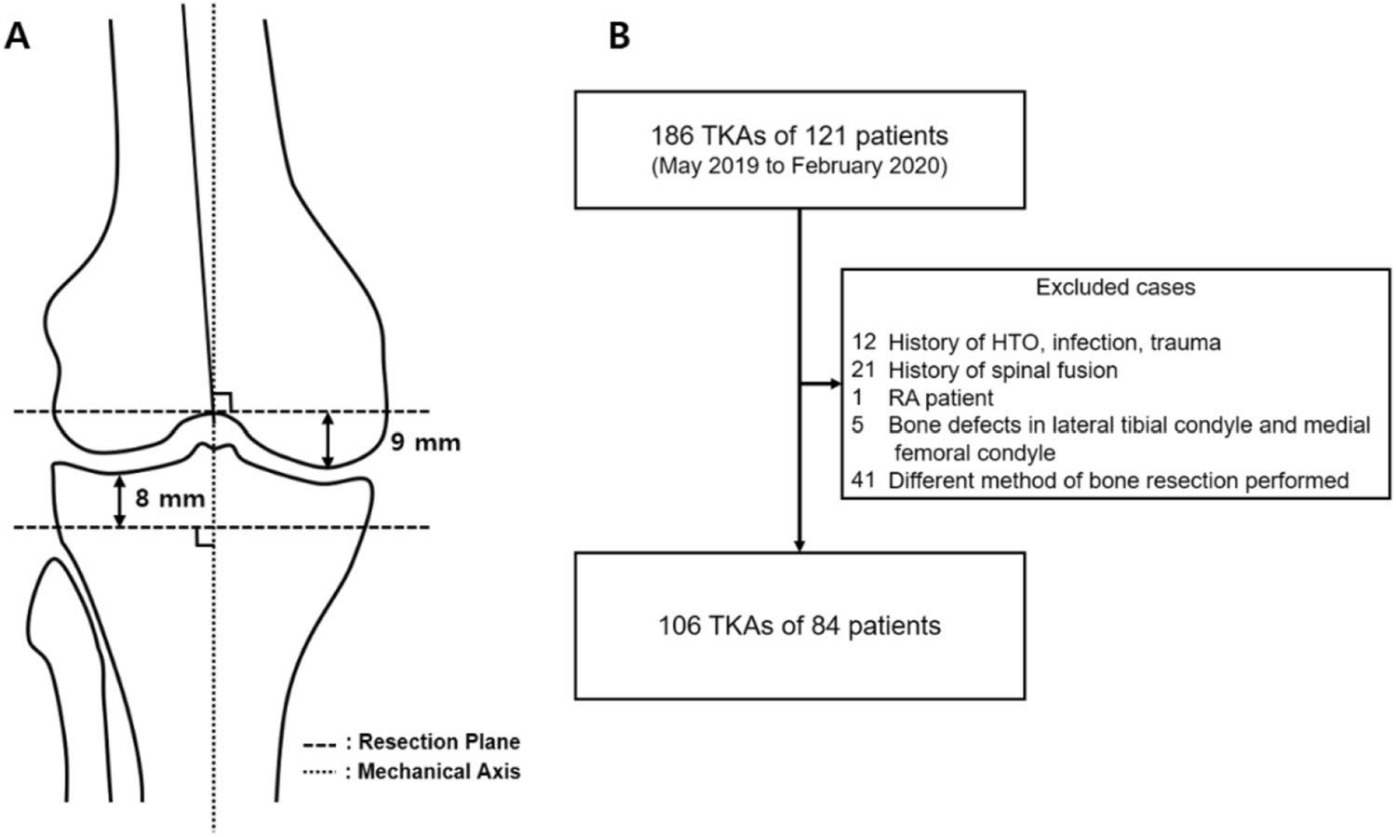
**(A)** The method of bone resection of patients enrolled in this study. **(B)** Flowchart of patient inclusion.

After receiving the approval from our Institutional Review Board, we retrospectively reviewed 186 knees of 121 patients who underwent primary TKA for degenerative osteoarthritis consecutively. Since the purpose of this study was to analyze the effect of soft tissue on the gap, only patients who underwent the same method of bone resection were included. The same method of bone resection was defined as follows: (1) distal femur resection was performed perpendicular to the femoral mechanical axis and at a distance of 9 mm from the most distal femoral condyle (mainly medial femoral condyle); and (2) proximal tibial resection was performed perpendicular to the tibial mechanical axis and at a distance of 8 mm from the most proximal tibial plateau (mainly lateral tibial condyle) (Fig. 1A). Exclusion criteria were as follows: (1) patients who did not undergo physical examinations or image studies; (2) patients who had a history of previous unicompartmental knee arthroplasty, high tibial osteotomy (HTO), infection, trauma, or rheumatoid arthritis; (3) patients who had a history of spinal fusion; (4) patients with bone defects in medial femoral condyle and lateral tibial condyle that cannot be used as a reference; (5) patients with motor weakness of lower extremity; and (6) patients who did not have bone resection, as mentioned above. A total of 80 TKAs were excluded for various reasons; and finally, a total of 106 TKAs of 84 patients were enrolled in this study (Fig. 1B). Demographic data and radiographic parameters are shown in Table 1.

**Table 1:** Demographics and baseline characteristics of patients.

	Mean ± SD (range) or %
Female sex (%)	74.5
Age (years)	72.1 ± 6.7 (56 to 87)
BMI (kg/m^2^)	25.8 ± 2.8 (19.0 to 36.9)
Anesthesia type (%)	
General/spinal	54.7/45.3
MPTA (°)	4.5 ± 3.0 (− 6.2 to 11.8)
EOS parameters	
Pelvic incidence (°)	53.6 ± 9.7 (32 to 88)
Sacral slope (°)	30.8 ± 10.3 (− 5 to 53)
Pelvic tilt (°)	21.9 ± 10.4 (− 8 to 50)
Preoperative hip-knee-ankle angle (°)	9.1 ± 6.3 (− 4.3 to 25.4)
Generalized joint laxity (%)	25.8
Kellgren-Lawrence grade (%)	
Grade 3/Grade 4	16.0/84.0
Flexion contracture angle (°)	7.7 ± 6.1 (0 to 30.0)
Active maximum flexion angle (°)	126.2 ± 12.6 (85.0 to 155.0)
Bilateral popliteal angle (°)	15.6 ± 7.2 (2.1 to 32.5)

*SD* standard deviation, *BMI* body mass index, *MPTA* medial proximal tibial angle, *BPA* bilateral popliteal angle.

### Popliteal angles and generalized joint laxity

**Figure 2:**
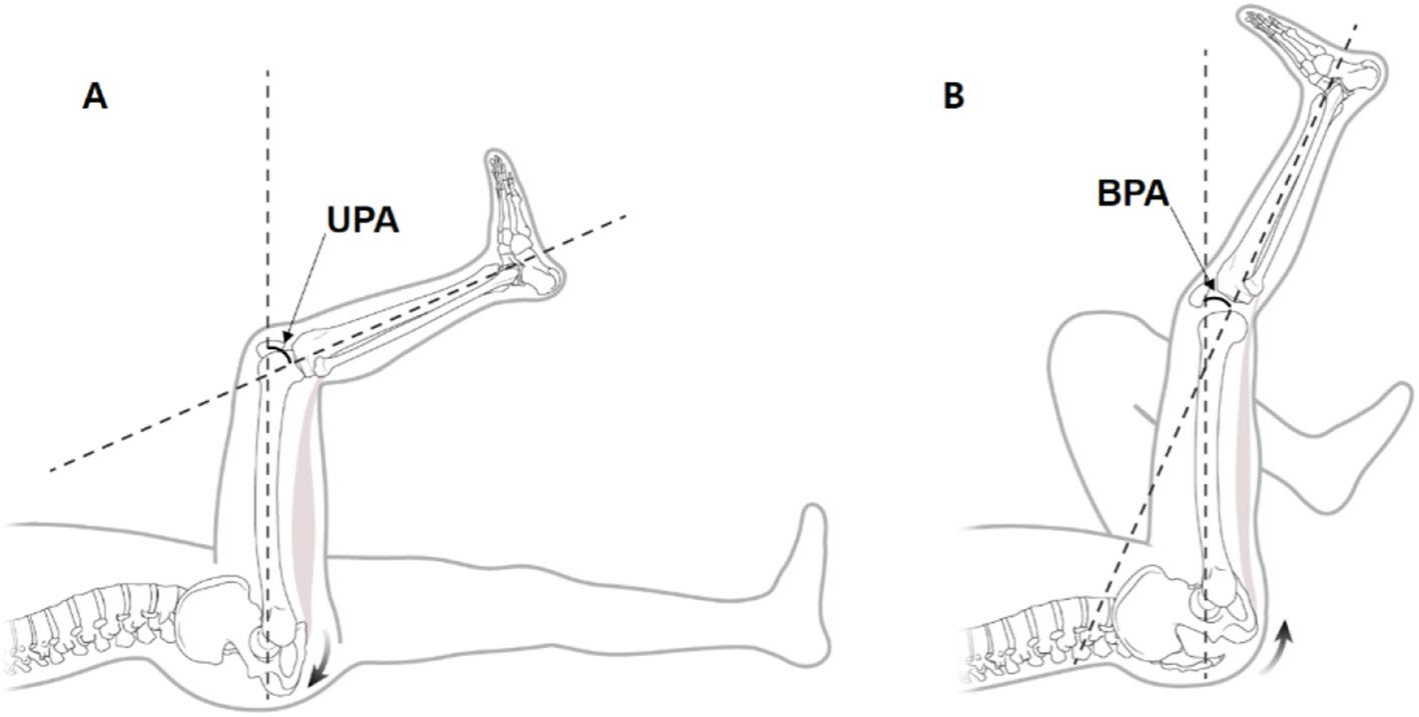
Popliteal angle was defined as the angle between the femoral extended line and tibia. **(A)** UPA was measured with the contralateral hips and knees extended. **(B)** BPA was measured with the contralateral hips and knees flexed to neutralize the anterior pelvic tilt.

Popliteal angles, range of motion (ROM), and generalized joint laxity of each patient were routinely measured to evaluate the soft tissue laxity. The flexion contracture measurements were made using a digital goniometer with reference to the lateral femoral epicondyle, greater trochanter, and lateral malleolus with the passively extended knee in the supine position of the patient. Popliteal angles are generally used to identify hamstring tightness in pediatric patients with cerebral palsy^6,7^. Popliteal angle was defined as the acute angle between tibia and femur when the knee joint was passively extended with the hip flexed 90 degrees. Unilateral popliteal angle (UPA) was measured with the contralateral hips and knees extended, indicating functional hamstring tightness (Fig. 2A). Bilateral popliteal angle (BPA) was measured with the contralateral hips and knees flexed to neutralize the anterior pelvic tilt, indicating true hamstring tightness (Fig. 2B). Among the two popliteal angles, Thomson et al*.* reported that the BPA neutralizing anterior pelvic tilting was an indicator of semimembranosus^8^. For this reason, our study used BPA as an indicator of hamstring tightness and excluded patients who had a history of spinal fusion, as there may be limitations of neutralization of pelvic tilt. Measurements were made with a digital goniometer similar to the flexion contracture measurements. Since the origin of hamstring muscles is located in the posterior-inferior aspect relative to the hip joint, hip joint flexion makes hamstrings tighter than extension; as a result, UPA is always larger than BPA.

The generalized joint laxity was also evaluated using the Beighton and Horan criteria^9^, which are widely used in the field of sports medicine. The Beighton and Horan criteria are as follows: (1) hyperextension of the little finger (5th digit) beyond 90°, (2) passive apposition of the thumb to the flexor aspect of the forearm, (3) hyperextension of the elbow beyond 10°, (4) hyperextension of the knee beyond 10°, and (5) forward flexion of the trunk so that the palms of the hands rest easily upon the floor. Items 1–4 are scored as either 1 or 0 for each side, right and left. The fifth item is scored as either 1 or 0. The total sum is the final score, ranging from 0 to 9. Generalized joint laxity is defined as a score ≥ 4 points. Higher scores indicate higher degrees of joint laxity. In the present study, none of the patients satisfied the fourth item due to arthritic flexion contracture.

### Gap measurement

**Figure 3:**
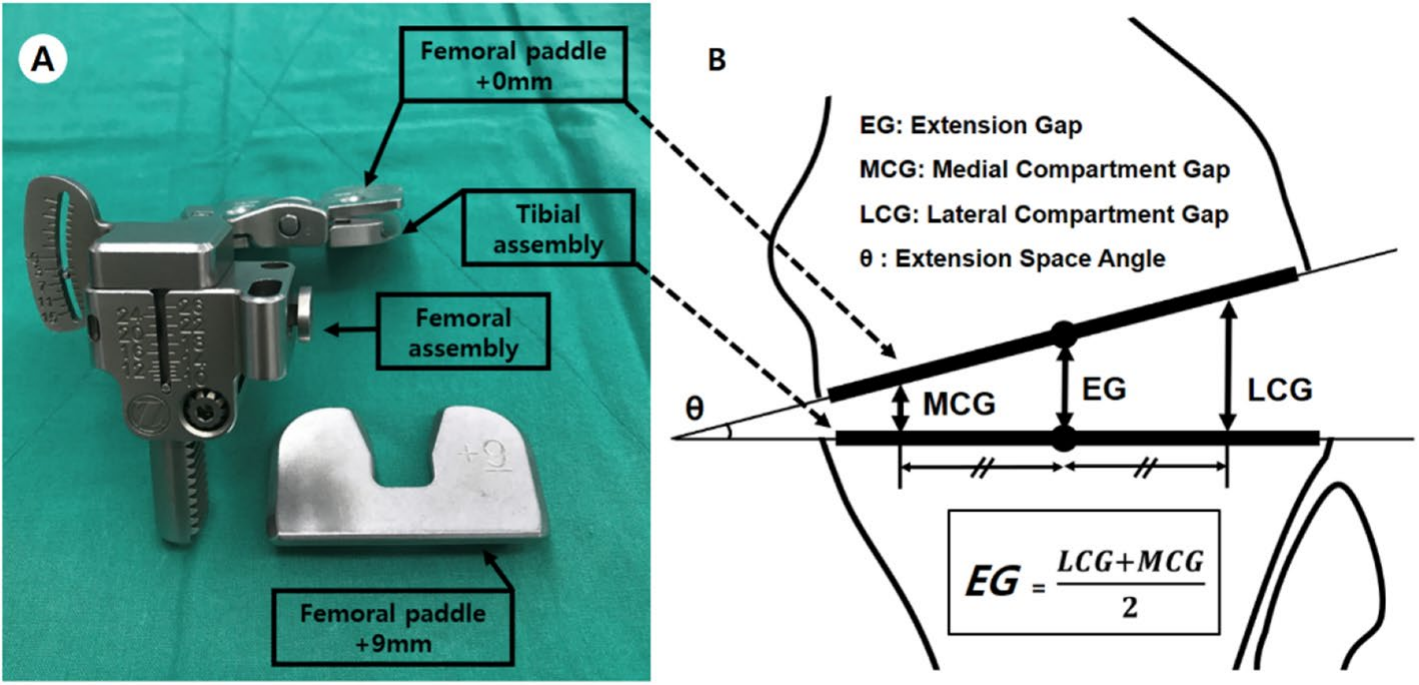
**(A)** Offset type tensor. **(B)** EG was defined as the distance between each center of tibial assembly and femoral paddle with distraction by the torque driver; and it was equal to the average value of LCG and MCG.

An offset type tensor was used in this study to measure the distance and angle between the resected bone planes. The offset type tensor FuZion (Zimmer Biomet, Warsaw, IN) makes it easy to measure the EG and extension space angle without the use of a navigation system. This device consists of three parts: femoral assembly, tibial assembly, and femoral paddle (Fig. 3A). The distance between the center of the femoral paddle and tibial assembly is the EG, and the angle between the two parts is the extension space angle. The EG is equal to the mean of the medial compartment gap (MCG) and the lateral compartment gap (LCG) (Fig. 3B). The offset type tensor was placed between the resection plane of distal femur and proximal tibia. Since the measurements could vary depending on the position of the offset type tensor, the anterior border of proximal tibia and tibial tuberosity were used as reference to obtain a constant position. Then, the EG was measured with patella reduction and temporary joint closure with towel clips. In the present study, joint distraction force was 40 lbf with toque driver.

### Surgical procedure

All patients underwent posterior stabilized TKA through the midvastus approach by one experienced surgeon (WSL) using the Persona Knee System (Zimmer Biomet, Warsaw, IN). All surgeries used the technique of mechanical alignment in TKA. After the removal of anterior cruciate ligament and posterior cruciate ligament, 9-mm resection of distal femur was performed perpendicular to the femoral mechanical axis using intramedullary guide. Since there was no varus femoral anatomy in this study, all distal femoral bone resection had been made from the medial femoral condyle. Then, proximal tibial resection was performed perpendicular to the tibial mechanical axis and with 3° of posterior slope at a distance of 8 mm from the least involved tibial condyle. In most patients, there were various forms of bone defects in medial tibial condyle, which made it difficult to set a consistent resection level from the medial tibial condyle. Therefore, tibial resection was performed based on lateral tibial condyle, and only the patients on which it was performed were enrolled in this study. The resection thickness of distal femur (9 mm) and proximal tibial (8 mm) did not include cartilage thickness. Afterward, EG and extension space angle were measured using the offset type tensor. At this time, only meniscectomy and deep medial collateral ligament release were performed, while other soft tissues that could affect the gap were not manipulated.

### Radiographic parameters

The Kellgren-Lawrence grade was evaluated using true anterior–posterior standing radiographs of the knee. The medial proximal tibial angles (MPTA) were evaluated by preoperative scanograms. As knee flexion compensates for the spinal sagittal imbalance^10^, we evaluated the pelvic parameters in elderly patients that might affect the tissue contracture around the knee. EOS imaging system^11^ was used to measure the pelvic parameters (pelvic incidence, sacral slope, and pelvic tilt) and hip-knee-ankle angle (HKA). Posterior slopes and tibial component coronal angle to anatomical axis were measured from postoperative radiographs for the evaluation of tibial bone resection.

### Statistical analysis

The associations between patient variables and EG were analyzed using multivariable linear regression and Pearson's correlation coefficients. The intra- and inter-observer reliability of measurements were assessed using intra-class correlation coefficients. Statistical analysis was performed using SPSS software for Windows (version 25.0; IBM Corp., Armonk, NY). The level of significance was set as p-value < 0.05. The statistical software G*Power (version 3.1.9.4; Heinrich Heine Universität Düsseldorf, DE) was used to calculate statistical power^12^. An effect size for statistical power calculation was obtained from the result of a linear regression analysis in a pilot study that included 40 subjects. Using α of 0.05 and the sample size of this study, the calculated statistical power was 0.99.

### Ethical approval

This retrospective study was approved by the Gangnam Severance Hospital Institutional Review Board (IRB # 3-2019-0228) and all methods were performed in accordance with the guidelines and regulations of Gangnam Severance Hospital IRB. A wavier of consent was obtained by Gangnam Severance Hospital IRB for retrospective collection and analysis for deidentified demographic and medical data.

## Results

**Figure 4:**
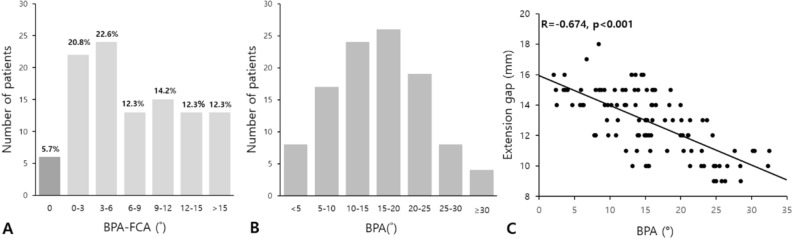
**(A)** Histogram for differences between BPA and flexion contracture angle (FCA). **(B)** Histogram for BPA divided into 7 categories by 5°. **(C)** Scatterplots of the correlation between EG and BPA (r = − 0.674, p < 0.001).

The average EG was 12.9 ± 2.1 mm, and the average extension space angle was 2.8° ± 3.2° (Table 2). BPA was greater than flexion contracture in most cases (94.3%), and no difference was found in only six cases (5.7%) (Fig. 4A). According to multivariable linear regression analysis which was conducted after modifying the BPA into a categorical variable by 5° (Fig. 4B), EG was correlated with BPA without variance inflation factor issue (p < 0.001) (Table 3). Pearson’s correlation coefficient between EG and BPA was − 0.674 (p < 0.001) (Fig. 4C). No other factors were significantly correlated with intraoperative EG.

**Table 2:** Intraoperatively and postoperatively measured parameters.

	Mean ± SD (range) or %
**Intraoperatively measured parameters**	
Extension gap (mm)	12.9 ± 2.1 (9 to 18)
Extension space angle (°)	2.8 ± 3.2 (− 4 to 14)
**Postoperatively measured parameters**	
Posterior slope (°)	3.0 ± 1.5 (0.2 to 6.8)
Tibial component coronal angle (°)	− 0.1 ± 1.3 (− 3.7 to 5.1)

*SD* standard deviation.

**Table 3:** Results of multivariable linear regression analysis for EG using categorical groups of BPA.

Variables	Univariable			Multivariable			
	B	SE	p-value	B	SE	p-value	VIF
Female sex	0.929	0.456	0.044*	0.374	0.444	0.401	1.36
Age (years)	− 0.011	0.031	0.710				
BMI (kg/m^2^)	0.066	0.071	0.359				
MPTA (°)	0.223	0.067	0.001*	0.086	0.079	0.281	2.50
Tibial component							
Coronal angle (°)	0.065	0.171	0.705				
Posterior slope (°)	0.090	0.156	0.568				
EOS parameters							
Pelvic incidence (°)	0.033	0.022	0.148				
Sacral slope (°)	0.012	0.021	0.572				
Pelvic tilt (°)	− 0.008	0.021	0.704				
Preoperative HKA (°)	0.169	0.028	< 0.001*	0.066	0.040	0.103	2.84
Beighton and Horan score	0.241	0.107	0.027*	0.070	0.088	0.429	1.25
Kellgren-Lawrence grade	0.835	0.547	0.130				
Anesthesia type	0.385	0.406	0.345				
Flexion contracture angle (°)	− 0.121	0.031	< 0.001*	− 0.017	0.033	0.611	1.60
Maximum flexion angle (°)	0.021	0.017	0.218				
BPA (categorical)	− 0.924	0.100	< 0.001*	-0.682	0.131	< 0.001*	1.59

Adjusted R^2^ = 0.523.

*B* regression coefficient, *SE* standard error, *VIF* variance inflation factor, *BMI* body mass index, *MPTA* medial proximal tibial angle, *HKA* hip-knee-ankle angle, *BPA* bilateral popliteal angle.

*p-value < 0.05.

Intra- and inter-observer reliability for the variables were as follows: 0.940–0.943 and 0.937–0.942 for ROM, 0.974 and 0.964 for BPAs, 0.901 and 0.850 for posterior slopes, 0.977 and 0.893 for tibial component coronal angle, 0.931 and 0.937 for MPTA at the 95% confidence interval, respectively.

## Discussion

The present study identified that BPA reflecting hamstring tightness was related to EG. The tighter the hamstring muscles, the smaller the EG. In addition, it was suggested that popliteal angle has its own meaning in the adult group without neuromuscular disease.

Flexion contracture is caused by a combination of bone impingement and soft tissue contracture^13,14^. Popliteal angle is similar with flexion contracture in view of the limitation of the knee joint extension, but the proportions affected by bone impingement and soft tissue contracture are different. If there is an osteophyte that induces flexion contracture in the posterior femoral condyle, the degree of soft tissue tenting is affected not only by the size of the osteophyte^15^, but also by the knee flexion angle. As the knee flexion angle is increased, posterior structures and their insertion sites move further backwards than the osteophyte^16^, so the effect of osteophyte decreases. Therefore, it can be considered that BPA, whose extension is restricted at an angle greater than flexion contracture angle, is less affected by osteophyte. According to Moon et al., the average flexion contracture was 1.0 (-0.1 to 2.5) and the average BPA was 24.6 degrees (18.7 to 30.3) in normal people aged 13–50 without OA^6^. The BPA value of this study was greater than that of the OA patient group in our study. If osteophyte had a greater effect on BPA, it would be more likely to have a greater BPA value in the OA group. Taken together, it can be concluded that the soft tissue laxity due to the distraction of the offset type tensor would have a greater correlation with BPA than with flexion contracture, and our multivariable linear regression results support this.

Anatomical structures of the posterior aspect of the knee resisted hyperextension and distraction force (gap measurement). Among these structures, the oblique popliteal ligament (OPL) was the most important structure to resist knee hyperextension biomechanically^17^. LaPrade et al*.* described OPL as one of eight distal attachments of the semimembranosus tendon and the largest structure over the posterior aspect of the knee^2^. Then, Thomson et al*.* reported that BPA is an indicator of short medial hamstrings (semimembranosus)^8^. In conclusion, BPA reflects the tightness of posterior semimembranosus complex, including its distal attachment OPL, and it seems to affect the EG.

The size of the EG itself, as well as the balance between extension and flexion gaps, are important in TKA. If the EG is too small, more bone resection is required, and this is not technically difficult^18,19^. However, if it is too large, thick bearings may be needed to avoid instability or recurvatum, and as such, surgeons should be more cautious about performing this procedure. According to Greco et al., the use of thick bearings equal to or larger than 16 mm does not have a higher failure rate compared to thin bearings^20^. However, large gaps with unnecessary bone resection may induce negative results. Adding distal femoral resection can result in joint line elevation^21^, and adding proximal tibial resection can lead to increased strain on the tibia^22^. In addition, it is necessary to minimize unnecessary bone resection, even in view of the possibility of future revisions^23^.

The Beighton and Horan criteria reflecting generalized joint laxity, which is also known to reflect the soft tissue laxity and joint hypermobility of the whole body, did not appear to affect the EG in elderly patients. Kwon et al*.* reported that two groups divided by generalized joint laxity showed difference in insert thickness^24^, but the choice of insert could be affected by the surgeon’s intentions, and this was not reproduced in this study. Unlike young patients, joint mobility of elderly patients is disrupted by joint degeneration and osteophyte apart from soft tissue laxity. To evaluate the generalized joint laxity, hyperextension of the knee joint should be measured. However, as most elderly patients with knee osteoarthritis tend to have flexion contracture, it is impossible to measure knee hyperextension. Instead, since popliteal angle is mostly measured before reaching the flexion contracture angle, it is advantageous to measure the soft tissue laxity around the knee joint in elderly patients.

This study had some limitations. First, there was a lack of previous studies on the significance of popliteal angle in adult patients. We tried to prove that the popliteal angle is an independent entity from flexion contracture in normal adults, but further study would be needed. Second, the results of this study may not be applied quantitatively, due to the differences in surgical methods and instruments used by different surgeons. However, this study is valuable in that it provides a rough trend of the EG in advance using a simple examination. Third, it was impossible to conduct a separate evaluation of collateral ligaments affecting the EG through physical examination alone. However, collateral ligaments are also stretched out by distractions like in the posterior structures^25^, so BPA seems to reflect not only the laxity of hamstring, but also the generalized soft tissue laxity around the knee. Just as the Beighton and Horan criteria is considered to reflect generalized joint laxity in the sports medicine field, hamstring tightness can reflect the soft tissue laxity around the knee including collateral ligaments in OA patients who cannot be applied the Beighton and Horan criteria, and statistical results of our study support this. Nevertheless, this study is still meaningful as it is the first to identify the relationship between hamstring tightness and extension gap. Moreover, it is the first to demonstrate that popliteal angle can be used as a method to measure hamstring tightness. Since BPA is a non-invasive and simple method, TKA surgeons would have no difficulty in applying this method for evaluation. Therefore, if BPA is performed preoperatively, it could be very helpful for both the surgery and the patient's prognosis, with only little effort.

## Conclusion

The present study found that popliteal angle is a different entity from flexion contracture, and that it is a predictable factor for EG in osteoarthritis patients. Smaller BPAs lead to larger EG among patients who underwent the same degree of bone resection. This finding would allow surgeons to predict the approximate soft tissue laxity and reduce unnecessary bone resection, thereby making it possible to determine an adequate EG.

## Data Availability

The datasets generated during and/or analysed during the current study are available from the corresponding author on reasonable request.
